# Benchmark Calculations for Perchlorate from Three Human Cohorts

**DOI:** 10.1289/ehp.7814

**Published:** 2005-04-20

**Authors:** Kenny S. Crump, John P. Gibbs

**Affiliations:** 1 Environ Health Sciences Institute, Ruston, Louisiana, USA; 2 Health Management Division, Kerr-McGee Shared Services LLC, Oklahoma City, Oklahoma, USA

**Keywords:** benchmark dose, perchlorate, reference dose, thyroid, thyroid-stimulating hormone, thyroxine

## Abstract

The presence of low concentrations of perchlorate in some drinking water sources has led to concern regarding potential effects on the thyroid. In a recently published report, the National Academy of Sciences indicated that the perchlorate dose required to cause hypothyroidism in adults would probably be > 0.40 mg/kg-day for months or longer. In this study, we calculated benchmark doses for perchlorate from thyroid-stimulating hormone (TSH) and free thyroxine (T_4_) serum indicators from two occupational cohorts with long-term exposure to perchlorate, and from a clinical study of volunteers exposed to perchlorate for 2 weeks. The benchmark dose for a particular serum indicator was defined as the dose predicted to cause an additional 5 or 10% of persons to have a serum measurement outside of the normal range. Using the data from the clinical study, we estimated the half-life of perchlorate in serum at 7.5 hr and the volume of distribution at 0.34 L/kg. Using these estimates and measurements of perchlorate in serum or urine, doses in the occupational cohorts were estimated and used in benchmark calculations. Because none of the three studies found a significant effect of perchlorate on TSH or free T_4_, all of the benchmark dose estimates were indistinguishable from infinity. The lower 95% statistical confidence limits on benchmark doses estimated from a combined analysis of the two occupational studies ranged from 0.21 to 0.56 mg/kg-day for free T_4_ index and from 0.36 to 0.92 mg/kg-day for TSH. Corresponding estimates from the short-term clinical study were within these ranges.

Perchlorate is one of several monovalent anions that have been shown to competitively inhibit thyroidal iodide uptake (Wyngaarden et al. 1952). At sufficiently high doses, perchlorate can block iodine uptake and thus inhibit thyroid function. This characteristic of perchlorate has been used in the treatment of Graves disease, a condition characterized by hyperthyroidism (Crooks and Wayne 1960; Godley and Stanbury 1954). The specific iodide transporter protein at which perchlorate competitively inhibits iodine uptake has been characterized (Dai et al. 1996).

The discovery of the widespread presence of low concentrations of perchlorate in drinking water in the southwestern United States (Urbansky 2002) has led to concern regarding the possibility that environmental perchlorate may induce a relative iodine deficiency and thereby decrease thyroid hormone production. If this were to happen during pregnancy among women with lower iodine intake, adverse neurodevelopmental outcomes in the fetus could result.

In response to these concerns, the U.S. Environmental Protection Agency (EPA) has undertaken to determine a reference dose (RfD) for perchlorate in drinking water. In 2003, the agency asked the National Academy of Sciences (NAS) to review the science supporting potential toxicity of perchlorate. An NAS committee recently reported their findings regarding the health implications of perchlorate ingestion (NAS 2005). In their report, the committee stated:

- “Inhibition of iodide uptake by the thyroid clearly is not an adverse effect.”
- “To cause declines in thyroid hormone production that would have adverse health effects, iodide uptake would most likely have to be reduced by at least 75% for months or longer.”
- “The committee does not agree that transient changes in serum thyroid hormone or TSH [thyroid-stimulating hormone] concentrations are adverse health effects; they are simply biochemical changes that might precede adverse effects.”
- “The committee concludes that the first adverse effect in the continuum would be hypothyroidism. Any effects that follow and result from hypothyroidism clearly would be adverse.”

The committee also concluded that “on the basis of the studies of long-term treatment of hyperthyroidism in which patients continued to be given perchlorate after their hyperthyroidism resolved . . . the perchlorate dose required to cause hypothyroidism in adults would probably be more than 0.40 mg/kg per day,” thus establishing a no observed adverse effect level (NOAEL) (NAS 2005).

The NAS further recommended an RfD of 0.0007 mg/kg-day based on a no observed effect level (NOEL) for iodine uptake inhibition provided by Greer et al. (2002) and a 10-fold uncertainty factor. They stated that this “value is supported by other clinical studies, occupational and environmental epidemiologic studies, and studies of long-term perchlorate administration to patients with hyperthyroidism.”

The U.S. EPA formally adopted the RfD of 0.0007 mg/kg-day recommended by the NAS committee. The agency defines an RfD as “an estimate (with uncertainty spanning perhaps an order of magnitude) of a daily oral exposure to the human population (including sensitive subpopulations) that is likely to be without deleterious noncancer effects during a lifetime” (U.S. EPA 2002). Given a body weight and a daily drinking water consumption, an RfD can be converted to an equivalent concentration in drinking water.

Traditionally, an RfD has been calculated by dividing a NOAEL by various safety factors. More recently a benchmark calculation has been used instead of a NOAEL in the determination of an RfD. A benchmark dose (BMD) is a dose corresponding to a prescribed increase in an adverse response (Budtz-Jørgensen et al. 2001; Crump 1984, 1995, 2002a, 2002b; Gaylor and Slikker 1990; Kodell et al. 1995; West and Kodell 1993). A statistical lower bound on the BMD (BMDL) replaces a NOAEL in determining an RfD (U.S. EPA 2000a, 2000b). A BMDL has several potential advantages over a NOAEL, including better reflection of the power of a study to detect effects, better use of dose response information from a study, and less dependence on the spacing of doses in a study. A BMDL can be calculated from data in which adverse responses occur at every dose, such that no NOAEL can be defined, and also from negative data in which there is no clear evidence of a dose-related effect (Crump et al. 2000).

Although an RfD can be calculated from either animal or human data, it is generally agreed that use of human data is appropriate whenever suitable human data are available. The NAS found that in the case of perchlorate, the animal data were not conclusive and that the human data are more than sufficient for risk assessment. They based their RfD on the NOEL for inhibition of radioactive iodine uptake (RAIU) from Greer et al. (2002), who studied thyroid function in volunteers given perchlorate in drinking water for 14 consecutive days. Two other studies of thyroid effects in humans exposed occupationally to perchlorate have been conducted that contain data suitable for supporting a benchmark analysis. Lamm et al. (1999) studied thyroid function of long-term ammonium perchlorate workers at a facility in Utah. Braverman et al. (2005) conducted a similar study of long-term workers from the same Utah facility. The workers typically worked three 12-hr shifts followed by 3 days off. Each of these studies evaluated various measures of thyroid function and either assigned perchlorate doses or collected serum or urine samples that could be used to quantify perchlorate exposure.

Not all of the different thyroidal end points that were evaluated in these studies are suitable to develop a BMDL. The various thyroidal measurements made included RAIU, thyroid volume, thyroglobulin (Tg), tri-iodothyronine (T_3_), total thyroxine (T_4_), free T_4_ (FT_4_), free T_4_ index (FTI), and TSH. T_3_ and T_4_ are the active thyroid hormones, and they can be either bound to serum proteins or free. Increases in Tg and thyroid volume are generally considered adaptive responses. FTI and FT_4_ are comparable quantitative measurements of non-protein-bound T_4_ in the serum.

The adverse outcome of most concern is a delay in T_4_-dependent neuronal cell migration during fetal development that occurs near the late first trimester and early second trimester (Lavado-Autric et al. 2003). At this critical early stage of pregnancy, the fetal thyroid is not yet functional, and the only source of fetal thyroid hormone is T_4_ that crosses the placenta from the maternal circulation. With maternal hypothyroidism, maternal T_4_ decreases significantly, resulting in a corresponding decrease of fetal thyroid hormone levels and also triggering an increase in maternal TSH.

Recent epidemiologic studies in Europe and the United States have concluded that maternal hypothyroidism during pregnancy, even when mild and considered subclinical, and especially when occurring during early gestation, may be associated with an impairment of normal brain development and intelligence in offspring (Haddow et al. 1999; Pop et al. 1999). Maternal FT_4_ and TSH are the critical thyroidal parameters that endocrinologists are focused on as measurable thyroidal end points of concern, although there remains uncertainty over which of these is most important.

Thus, to cause an adverse neurodevelopmental effect in the fetus, perchlorate would have to be present at levels sufficient to cause hypothyroidism and thus cause a measurable decrease in maternal T_4_ or increase in maternal TSH. In the present report, we calculated BMD using FT_4_ and TSH measurements in conjunction with calculated or measured perchlorate doses from these three studies. These calculations are compared with the 0.4 mg/kg-day NOAEL estimate from the NAS.

## Materials and Methods

### Human Studies

#### Study of ingestion of perchlorate by volunteers.

Greer et al. (2002) gave perchlorate in drinking water to 37 male and female volunteers for 14 consecutive days. This study is likely too short in duration to find significant changes in FT_4_ and TSH, based on the NAS (NAS 2005) statement that “iodide uptake would most likely have to be reduced by at least 75% for months or longer.” Nevertheless, it may be useful to calculate a BMDL for short-term exposure.

In the main study, four subjects of each sex received a perchlorate dose of 0.02, 0.1, or 0.5 mg/kg-day. Subsequently, six women and one man received a dose of 0.007 mg/kg-day and one additional subject of each sex received a dose of 0.02, 0.1, or 0.5 mg/kg-day (the uptake study). All participants were instructed to drink one-fourth of a mixture containing a body-weight-adjusted daily dose at 0800, 1200, 1600, and 2000 hr on each scheduled perchlorate ingestion day and to record the time and volume of each ingestion.

Greer et al. (2002) collected blood samples during the main study at the screening visit held 6 days before the beginning of exposure, on the day before the beginning of exposure (0800 hr), exposure day 1 (1200 and 1600 hr), exposure day 2 (0800, 1200, and 1700 hr), exposure day 3 (0800 hr), exposure day 4 (0800 and 1200 hr), exposure day 8 (0800 hr), exposure day 14 (0800, 1200, and 1700 hr), postexposure day 1 (0900, 1200, and 1700 hr), postexposure day 2 (0800 and 1700 hr), post-exposure day 3 (0800 and 1700 hr), postexposure day 4 (0800 hr), and postexposure day 14 (0800 hr). Serum perchlorate was measured in most of these 23 sampling times, and critical thyroid function measurements (FT_4_ and TSH) were obtained at 16 of these times.

In the uptake study, serum perchlorate was not measured, and thyroid function measurements were made only from blood samples collected at the screening visit and on exposure day 14 (0800 hr). The precise time of each sample collection was recorded in both studies. Also, RAIU measurements and urine samples were collected at various times in both studies.

Greer et al. (2002) found iodine uptake to be inhibited at the three highest doses but found no association between dose and indicators of thyroid function, other than a slight reduction in TSH levels in morning blood draws during perchlorate exposure within the highest exposure group (0.5 mg/kg-day), with recovery during 15 days of postexposure. This downward trend is opposite the effect expected if it were due to perchlorate, although TSH can be decreased in regions with iodine deficiency.

#### Study of ammonium perchlorate workers: I.

Lamm et al. (1999) conducted a cross-sectional study of workers engaged in the production of either ammonium perchlorate (*n* = 37) or sodium azide (control workers, *n* = 21) in the same industrial complex. The workers worked 12-hr shifts on 3 consecutive days followed by 3 consecutive days off. They rotated shifts from days to nights monthly. Forty percent of the perchlorate production workers and 50% of the control workers had been employed for > 5 years.

Lamm et al. (1999) measured urinary perchlorate in each worker at the beginning and at the end of one shift. Postshift blood samples were obtained from all but one worker. The critical thyroid parameters measured were serum FTI and TSH. A perchlorate elimination half-life of 8 hr was estimated by monitoring urinary perchlorate in two of the more highly exposed workers for 3 subsequent days in which there was no known perchlorate exposure. The absorbed perchlorate dose (milligrams per shift) of each worker was then estimated by applying a first-order elimination model to the pre- and postshift measurements of perchlorate per gram of creatinine, assuming a creatinine excretion rate of 1 mg/min and a perchlorate elimination half-life of 8 hr.

Workers were grouped into four exposure categories with mean absorbed perchlorate dosages of 1, 4, 11, and 34 mg of perchlorate per day. No differences in thyroid function parameters were found between these groups.

#### Study of ammonium perchlorate workers: II.

Braverman et al. (2005) conducted a cross-sectional study of 29 workers employed in the same ammonium perchlorate production facility that was studied earlier by Lamm et al. (1999). Twelve community controls were also studied. The minimum duration of employment in ammonium perchlorate manufacture among the workers was 1.7 years, and the median was 5.9 years. This study was conducted approximately 6 years after the earlier study, and several workers were included in both study cohorts.

The workers worked the same shift pattern (12 hr on, 12 hr off for 3 consecutive days, followed by 3 days off) as in the Lamm et al. (1999) study. The study period for each worker encompassed work shifts on 3 consecutive nights, Tuesday through Thursday. Among the employees with the highest perchlorate exposure, RAIU was decreased 75% or more after 3 consecutive 12-hr shifts.

Urine and blood samples were collected from workers on Tuesday morning before their first night shift and at the beginning and end of the Thursday night shift. Community controls had their blood and urine sampled once, on Tuesday morning. Tuesday and Friday morning serum samples were analyzed for TSH, FTI, and perchlorate. Thursday evening serum samples were analyzed for perchlorate only. Creatinine and perchlorate were measured in all urine samples.

Serum FTI was slightly but significantly increased in the workers on Friday morning compared with Tuesday morning, and TSH was lower on Tuesday morning in perchlorate workers than in community controls. Both of these responses are opposite what would be expected from perchlorate exposure. Otherwise, there were no statistically significant differences in FTI or TSH in perchlorate workers between results on Tuesday and Friday mornings, or between perchlorate workers and community controls on Tuesday morning.

### Methods

#### Determination of serum half-life and volume of distribution.

To estimate perchlorate exposures in the Braverman et al. (2005) occupational study, we need estimates of the half-life, *t*_1/2_, and volume of distribution, *V*_*d*_, of per-chlorate. These parameters were estimated using the Greer et al. (2002) study of perchlorate uptake in volunteers.

The elimination of perchlorate from serum was assumed to be a first-order process with time constant β = ln(2)/*t*_1/2_. The movement of perchlorate from the gut into the systemic circulation was described by active transport with time constant α. If at time *t*_0_ an amount *D* (milligram per kilogram body weight) of perchlorate is ingested, the resulting serum concentration of perchlorate at time *t*, *C*(*t*) (milligram per liter), satisfies the differential equation


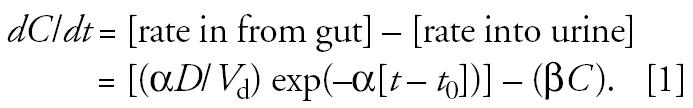


Solving, the contribution to serum by perchlorate ingested at time *t*_0_ is


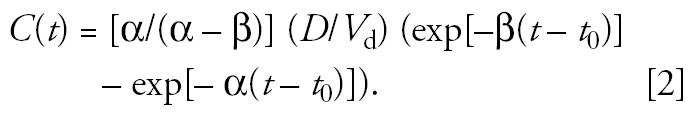


By summing the contributions from each ingestion time, *t*_0_, the total predicted serum concentration at each serum sample time, *t*, in the Greer et al. (2002) study was calculated.

To estimate α, β, and *V*_*d*_, the model defined by Equation 2 was fit to the Greer et al. (2002) serum perchlorate data assuming that the serum measurements (either the serum perchlorate concentrations or their log-transforms) can be modeled as *E_i,j_* + τ*_i_* + ɛ*_ij_*, where E*_i,j_* is the measurement predicted by the above model (or its log-transform) for the *i*th subject at the *j*th measurement time, and τ*_i_* and ɛ*_ij_* (where *i* and *j* index subject and measurement time, respectively) are independent and normally distributed with zero mean, τ*_i_* having standard deviation, σ_τ_, that represents interindividual variability, and ɛ*_ij_* having standard deviation, σ_ɛ_, that represents measurement error and temporal variability. It follows that the variance of a serum measurement is σ_τ_^2^ + σ_ɛ_^2^, and the correlation between two measurements is σ_τ_^2^/(σ_τ_^2^ + σ_ɛ_^2^) if the measurements are from the same individual, and zero otherwise.

#### Estimation of absorbed doses in Braverman et al.

We used the values for serum half-life, *t*_1/2_, and volume of distribution, *V*_*d*_, estimated from the Greer et al. (2002) study to estimate the perchlorate exposure for each worker in the Braverman et al. (2005) occupational study. We obtained two sets of estimates, one based on serum concentrations and one based on urine concentrations. Retaining the assumption that the elimination of perchlorate from serum is a first-order process, and assuming that inhaled perchlorate taken up by the lungs immediately enters the systemic circulation, the concentration (mg/L) in serum at the end of a 12-hr shift is


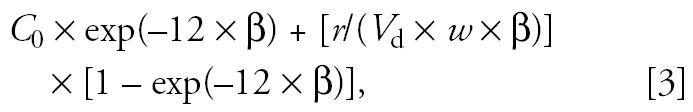


where *C*_0_ is the serum concentration (mg/L) at the beginning of the shift, *r* is the uptake rate of perchlorate (milligrams per hour, assumed constant throughout a shift for a given worker), and *w* is body weight (kilograms). Summing terms of this form for each of the first two shifts yielded an expression for the predicted concentration at the beginning of the third shift that was set equal to a worker’s measured serum concentration and solved for the uptake rate, *r*, for the first two shifts. Using this value of *r*, the concentration predicted at the beginning of the third shift was used as *C*_0_ in Equation 3, and the resulting predicted concentration at the end of the third shift was set equal to the measured serum level to determine a worker’s uptake rate, *r*, associated with the third shift.

We applied a conceptually similar approach to the perchlorate urinary concentrations (milligrams per gram creatinine) measured at the beginning and end of the third shift to estimate the uptake rate for the third shift based on the urine data. This analysis assumed the serum half-life, *t*_1/2_, estimated from the Greer et al. (2002) study and a constant creatinine elimination rate of 1 mg/min for all workers. This approach requires knowledge of the time since the most recent bladder void, which was not recorded. Different values of this time ranging from zero to 12 hr were considered.

#### Data used for benchmark calculations.

BMD calculations for the Lamm et al. (1999) study used postshift serum TSH and FTI measurements from both control and perchlorate workers. Calculations for the Braverman et al. (2005) study used TSH and FTI measurements collected from perchlorate workers and community controls on Tuesday morning, just before the Tuesday–Thursday night shifts. In both cases, the dose used in the calculation was a worker’s estimated average daily dose (milligrams), calculated as one-half of his estimated shift dose (reflecting the 3-day-on/3-day-off pattern of work).

BMD calculations for the Greer et al. (2002) study used TSH and FT_4_ data obtained from serum collected at the screening visit, on the day before the beginning of exposure, and on the last day of exposure (exposure day 14). To reflect to the fullest any effect of perchlorate exposure, measurements from serum collected after exposure began but before the last day of exposure were not used. The perchlorate dose (milligrams) assumed for serum collected on exposure day 14 was the assigned daily dose (0.007, 0.02, 0.1, or 0.5 mg/kg), multiplied by a subject’s body weight.

#### Statistical analysis.

In modeling the FTI and TSH data from Lamm et al. and Braverman et al. cohorts, it was assumed that a thyroid measurement, or its log-transform, was normally distributed with mean


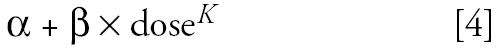


and constant SD, σ, where dose is the daily perchlorate dose in milligrams.

When applied to the Greer et al. (2002) FT_4_ and TSH data, we expanded the model defined by Equation 4 in two ways. First, an error structure was assumed like that applied to the Greer et al. (2002) serum perchlorate data (described above) that accounted for repeated measurements made in the same individual. Second, to account for potential daily variation in FT_4_ and TSH, the parameter α was allowed to assume different values depending on the time of day a sample was collected (before 1000 hr, between 1000 hr and 1400 hr, and after 1400 hr).

Unless otherwise specified, we estimated model parameters using maximum likelihood, hypothesis tests were likelihood ratio tests, and we computed 90% confidence intervals (90% CIs) by the profile likelihood method (Cox and Hinkley 1974; Crump 2002b; Venzon and Moolgavkar 1988). Residuals from model fits to the untransformed serum measurements were tested by the Shapiro-Wilk test for conformity with a normal distribution (Shapiro and Wilk 1965). If normality was rejected (*p* < 0.05), the analysis was repeated using the log-transform of the serum measurements. If the data were still non-normal, any outliers present were omitted (an outlier defined as a measurement whose residual was more than three times the SE) and the data retested for normality. The reported analysis was the one in which the residuals conformed most closely to normality. Because the potential adverse effect of perchlorate is an increase in TSH or a decrease in T_4_, the maximum likelihood estimate of β was not allowed to be negative when calculating BMDLs for TSH and not allowed to be positive when calculating BMDLs for FTI or FT_4_. To avoid biologically implausible dose–response curve shapes, the shape parameter, *K*, was not allowed to be < 1.0 (Crump 2002a) and, for computational reasons, was not allowed to exceed 10. Because there was no significant effect of dose on any thyroid parameter, *K* was allowed to differ from 1.0 only in BMD calculations. All calculations were performed in Excel (version 2002 SP3; Microsoft Corporation, Redmond, WA). Excel Visual Basic macros were used to calculate likelihoods, and parameters were estimated by maximizing the likelihood using the Excel optimizer routine.

#### Benchmark analysis.

A BMD is a dose corresponding to a specified change in response. For binary data coded as presence or absence of disease, the response is usually defined as the probability of disease. For continuous data, such as serum thyroid measurements, there is less agreement regarding how the BMD should be defined.

In the present analysis, the BMD is defined as the change in the mean serum measurement equal to a factor, *Q*, times the SD of the measurements. It follows from this definition and Equation 4 that BMD = (*Q* × σ/β)^1/^*^K^*. BMD calculations are presented using three different values for *Q*: 1.0, 0.82, and 0.52. In its benchmark technical guidance document, the U.S. EPA recommends that this method with *Q* = 1 (termed the “SD approach”) always be among the methods applied (U.S. EPA 2000a). The other two values, *Q* = 0.82 and *Q* = 0.52, are derived from the equivalent “hybrid” approach for defining a BMD, as explained below.

In the “hybrid” approach for defining a BMD from a continuous response, the proportion *P*(0) of unexposed individuals whose response is considered adverse is first specified, and the BMD is defined as the dose that increases the probability of an adverse response by a specified amount termed the benchmark risk (BMR) (Budtz-Jørgensen et al. 2001; Crump 1995; Crump et al. 2000; Gaylor and Slikker 1990; Kodell et al. 1995; West and Kodell 1993). This approach is conceptually similar to that generally applied to binary responses, and consequently its use provides comparability between BMR calculated from continuous and binary data. It can be shown that the hybrid approach, when implemented using Equation 4, is equivalent to the method described above in terms of *Q*, with


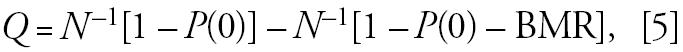


where *N*^–1^ is the inverse of the standard normal distribution (Budtz-Jørgensen et al. 2001; Crump 2002a).

The convention in evaluating serum thyroid clinical measurements is that the normal range includes the measurements of 95% of a normal (unexposed) population, and that abnormal values include 2.5% of the lowest measurements and 2.5% of the highest measurements. Because an adverse effect of perchlorate would be expected to result in decreased FTI or FT_4_ and increased TSH, *P*(0) was set to 0.025. Two values of BMR were used with the hybrid method: 0.05 and 0.1. BMR = 0.1 has often been used by the U.S. EPA when calculating a BMD from binary data, and BMR = 0.05 represents a more conservative choice. From Equation 5, when combined with the choice *P*(0) = 0.025, BMR = 0.1 is equivalent to *Q* = 0.82 and BMR = 0.05 is equivalent to *Q* = 0.52.

Ideally, the BMD calculation should reflect interindividual variation but not measurement error. In most data sets, it is not possible to separate contributions to variation from these two sources. However, in the Greer et al. (2002) study it is possible to separately estimate interindividual variation because repeated measurements were collected from each individual. Consequently, in benchmark calculations based on the Greer et al. data, the standard deviation for interindividual variability, σ_τ_, was used in place of the total standard deviation, σ = (σ_τ_^2^ + σ_ɛ_^2^)^1/2^.

Each of the data sets from which BMD calculations were made was negative; that is, there was no statistically significant evidence of a relationship between perchlorate dose and thyroid function. Even if there is apparently no effect of dose, a BMDL calculated from such negative data still represents a valid lower bound on the dose corresponding to any (undetected) effect of perchlorate that may have been present. There are a number of examples in the literature where a BMD analysis has been applied to data in which no adverse effects were detected (Clewell et al. 2000, 2003; Crump et al. 2000; NAS 2000).

Because the Lamm et al. (1999) and Braverman et al. (2005) studies were conducted in the same facility and made comparable measurements of thyroid function and perchlorate dose, we conducted a combined benchmark analysis of these two data sets. In this analysis the basic model (Equation 4) was expanded to account for potential differences between the two studies in laboratory techniques for measuring FTI and TSH, and for potential drift in normal thyroid function values. In the expanded model, the responses in one study were assumed to differ from the other, on average, by a fixed factor. This was accomplished by retaining Equation 4 to model the Lamm et al. data, but in modeling the Braverman et al. data multiplying both the mean (Equation 4) and the standard deviation, σ, by the same free parameter. The BMD as defined herein is functionally independent of this parameter.

The BMDL was calculated as a 95% statistical lower bound on the BMD in all instances.

## Results

### Serum half-life and volume of distribution.

In fitting the perchlorate uptake and elimination model (Equation 1) to the Greer et al. (2002) serum perchlorate data, we included only data from subjects in the main study exposed to the two highest doses (0.5 and 0.1 mg/kg/day). Serum perchlorate was not measured in the uptake study, and perchlorate was below the level of detection in most (58 of 62) of the serum measurements in subjects exposed to 0.02 mg/kg-day. Ninety-one additional samples in which no perchlorate was detected were also omitted. All but three of these were collected after dosing had ceased. One of these three appears to be an error, and the remaining two were collected from a single individual in the 0.1 mg/kg-day exposure group during the first day of exposure. After these exclusions, there remained 238 serum perchlorate measurements from the Greer et al. study available for analysis.

**Figure 1:**
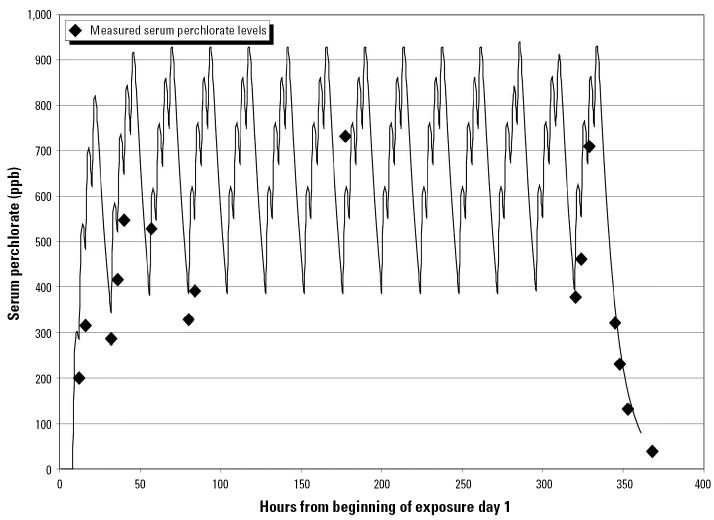
Serum perchlorate measurements for a subject from Greer et al. (2002) dosed at 0.5 mg/kg/day, versus expected concentrations.

The log-transformed serum concentrations were described much better by a normal distribution than by the untransformed concentrations. After elimination of four outliers, the residuals from fitting the log-transform of the model (Equation 1) to the log-transforms of the concentrations were marginally well described by a normal distribution (*p* = 0.04). Figure 1 shows, as an example, the serum concentration data for a subject in the 0.5 mg/kg-day dose group compared with the concentration profile predicted by the best-fitting model. The concentration spikes resulting from each of the four ingested doses on each of the 14 dosing days are visible in this graph. The peak serum concentration is predicted to have occurred at about 2200 hr each evening, a couple of hours after the last scheduled ingested dose for the day, and several hours after any scheduled serum measurement.

Based on this analysis, the half-life of perchlorate in serum was estimated as *t*_1/2_ = 7.5 hr (90% CI, 7.2–7.8) and the volume of distribution as *V*_*d*_ = 0.34 L/kg (90% CI, 0.31–0.39). The estimated between-subject and within-subject SD were σ_τ_ = 0.24 and σ_ɛ_ = 0.25. Estimates of *t*_1/2_ and *V*_*d*_ changed only slightly (by at most 8%) when outliers were not removed or when a normal distribution was assumed.

Using the best estimates from Greer et al. (2002) for the elimination half-life (*t*_1/2_ = 7.5 hr) and volume of distribution (*V*_*d*_ = 0.34 L/kg) for perchlorate, and Equation 3, we estimated the average per-shift dose (milligrams per shift) of each worker in the Braverman et al. (2005) study from the serum perchlorate measurements. Separate estimates were obtained for the first two shifts combined and for the third shift, and a weighted average of these two estimates was used as the average dose per shift for a worker.

**Figure 2:**
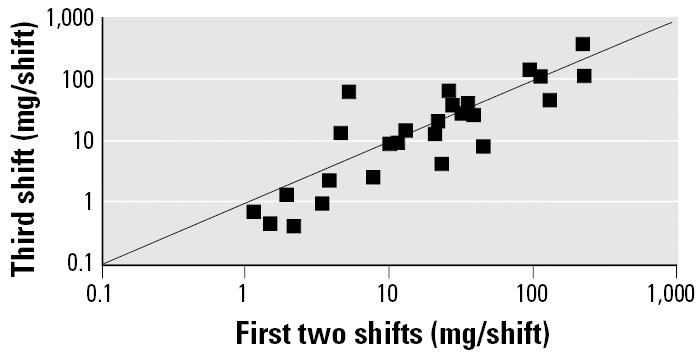
Comparison of shift doses in Braverman et al. (2005): average dose in first two shifts compared with third-shift dose.

Figure 2 compares the estimated per-shift dose for the first two shifts with the estimated dose for the third shift. Although there is a wide range in individual doses, from 0.41 mg/shift to 392 mg/shift, the difference in individual shift doses between the first two shifts and the third shift are relatively small in most cases, which supports the assertion that workers tended to work in similar jobs on different shifts.

**Figure 3:**
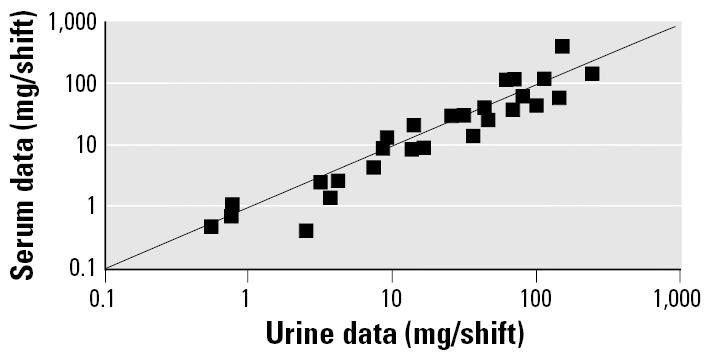
Comparison of shift doses in Braverman et al. (2005): third-shift doses estimated from urine or serum data.

Figure 3 compares estimates of dose during the third shift computed using urine data with those computed using serum data. The estimates based on the urine data assumed that the data are from a 4-hr void. By comparison, if a theoretical “instantaneous” void was assumed, these estimates were about 10% smaller, and if a 12-hr void was assumed, they were about 50% larger. Because the dose estimates based on the urine data were only for the third shift, and because of the uncertainty in both the void time for the urine samples and the rate of creatinine elimination, the average dose per shift over the three shifts obtained from the serum data were used in the benchmark analysis.

### Benchmark results.

In the Lamm et al. (1999) study, the residuals of the FTI were adequately described by a normal distribution (*p* = 0.38), and after eliminating one TSH measurement that was four times larger than that of any other worker, the residuals from the log-transformed TSH measurements were also normal (*p* = 0.12). In Braverman et al. (2005), both the residuals of the FTI measurements and the log-transformed TSH measurements were adequately described by a normal distribution (*p* = 0.54 and 0.16, respectively). Consequently, BMD analyses of FTI from these studies used the untransformed measurements, whereas analyses of TSH used the log-transformed measurements.

The model fit to the combined Lamm et al. (1999) and Braverman et al. (2005) data sets contained four estimated parameters, compared with the total of six that were estimated from the separate fits to the two data sets. By adding the log-likelihoods from the individual fits, we determined that the combined individual fits were not significantly better than the fit of the single model to the combined data (*p* = 0.52 and 0.98, 2 df, for FTI and TSH, respectively). The multiplicative factors needed to adjust the Braverman et al. results to conform to the Lamm et al. results were 0.38 for FTI values and 0.85 for the logarithms of TSH.

The estimated average daily exposure of one worker from the Braverman et al. cohort was 84 mg/day, which was more than twice that of any other worker. Although this worker’s FTI and TSH values were not exceptional, the BMDLs were highly dependent on this single data point. Consequently, BMDLs were calculated both with this influential point included and with it omitted.

**Figure 4:**
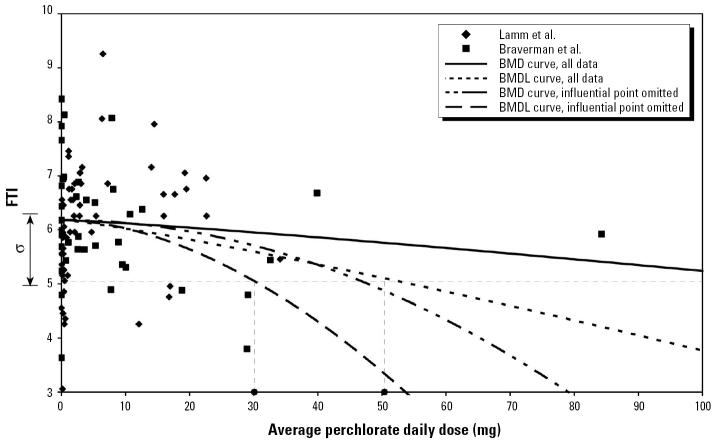
FTI data from Lamm et al. (1999) and scaled FTI data from Braverman et al. (2005) versus estimated daily perchlorate dose, with graphical indication of the BMDL calculation for *Q* = 1 (BMD defined as change in mean response equal to SD).

**Figure 5:**
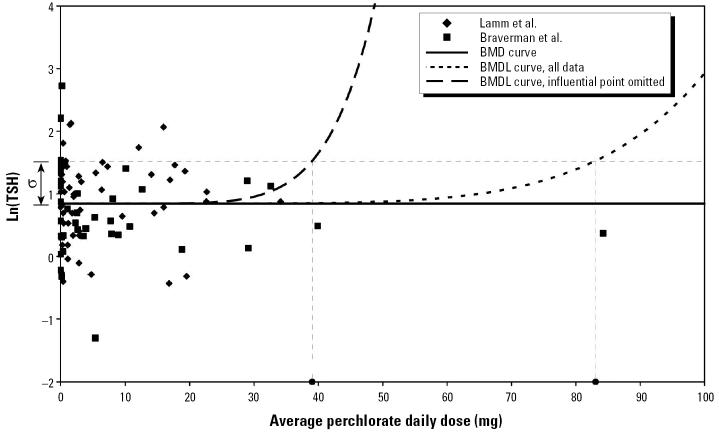
Log-transformed TSH data from Lamm et al. (1999) and scaled FTI data from Braverman et al. (2005) versus estimated daily perchlorate dose, with graphical indication of the BMDL calculation for *Q* = 1 (BMD defined as change in mean response equal to SD).

Figure 4 shows plots of the FTI measurements from Lamm et al. along with the adjusted FTI measurements from Braverman et al. Figure 5 shows comparable plots for the logarithm of TSH. These figures also show the maximum likelihood model fits that define the BMD (termed “BMD curve” in the figures) and the model curves that define the BMDL for the case *Q* = 1, both using all data and with the influential point omitted. The calculation of the BMDLs for *Q* = 1 are illustrated graphically.

Table 1 contains the BMD and BMDL estimates obtained from the combined data from the two occupational studies using all three values of *Q*, both including and omitting the influential data point. Also shown are the values of the shape parameter, *K*, corresponding to each BMDL calculation. The BMD estimates for TSH were infinite, because in both cases the maximum likelihood estimate of the parameter β was zero. Nevertheless, the corresponding BMDLs were all finite. The six calculations of BMDL based on all the data ranged from 24 to 83 mg/day or, by dividing by the average body weight in the study of 90 kg, from 0.27 to 0.92 mg/kg-day, and those with the influential point eliminated ranged from 16 to 39 mg/day, or from 0.18 to 0.43 mg/kg-day.

Turning now to the Greer et al. (2002) study, after elimination of two outliers, residuals from fitting the benchmark model to the remaining 134 FT_4_ measurements from this study were adequately described by a normal distribution (*p* = 0.11). Likewise, after elimination of two outliers, residuals from fitting the model to the log-transforms of the remaining 137 TSH measurements were normally distributed (*p* = 0.49). Time of day was a significant predictor of TSH (*p* = 0.0002), but not of FT_4_ (*p* = 0.24), with TSH measurements made before 1000 hr being larger than those made later in the day, but with no difference between midday and afternoon measurements. Consequently BMD analyses for FT_4_ did not allow for differences in time of day, whereas those for TSH permitted morning measurements to be different from those made later in the day. TSH was significantly decreased in serum collected at the conclusion of the 14-day exposure period (*p* = 0.002), whereas the expected effect of perchlorate would be to increase TSH.

Table 2 summarizes the benchmark calculations made from the Greer et al. (2002) short-term study. The BMD analyses for TSH assumed that an increase in TSH was adverse, despite the fact that a significant decrease was observed. The BMDLs from this study are all in a relatively narrow range, between 49 and 60 mg/day or, after dividing by the average body weight in the study of 76.4 kg, between 0.64 and 0.79 mg/kg-day, which is within the range of the BMDLs obtained from the occupational studies (Table 1).

## Discussion

The Lamm et al. (1999) and Braverman et al. (2005) studies were conducted 6 years apart in the same plant on overlapping cohorts. The same types of serum measurements were collected in the two studies. A single model that accounted for potential differences in serum thyroid measurement techniques, and for potential drift in normal thyroid function values, fit the combined data statistically as well as the separate models fit the individual data sets. The BMDLs from this combined analysis ranged from 0.18 to 0.56 mg/kg-day for FTI and from 0.36 to 0.92 mg/kg-day for TSH. The BMDLs from the Greer et al. (2002) study were within the range of those from the two occupational studies.

None of the three studies found evidence of an effect of perchlorate on thyroid function. Nevertheless, BMDLs calculated from such negative results represent valid statistical lower bounds on the dose that accounts for a potential, but unobserved, effect of perchlorate. However, BMDLs such as these based on negative data could possibly be highly conservative (Clewell et al. 2000; Crump et al. 2000).

Forty percent of the perchlorate workers in the Lamm et al. cohort had worked at the facility for > 5 years, and 50% of the workers in the Braverman et al. cohort had worked for at least 5.9 years. Based on jobs assigned to workers during the previous year and estimated perchlorate exposures in each job, Braverman et al. (2005) estimated a median shift dose over the previous year of 0.33 mg/kg. Therefore, the BMDLs obtained from the combined analysis of the two occupational studies pertain to exposures extending to 5 years or more in duration. Overall, the BMDLs calculated from the occupational studies are in excellent agreement with the NOAEL of 0.4 mg/kg-day obtained by the NAS based on clinical studies (NAS 2005).

In addition to BMDLs from the combined occupational cohorts, BMDLs were also computed from the two studies individually (results not shown). As expected, BMDLs from the combined data were larger than the smallest BMDLs from the individual data sets and tended to be either intermediate between those calculated from the individual data sets, or somewhat larger than the larger of the two BMDLs obtained from the individual data sets. Given that the two occupational studies had similar designs, that they were conducted in the same facility, and that the data were compatible with a single model, we believe the combined analysis makes the most appropriate use of the data from these studies.

The BMD calculations for the Braverman et al. (2005) cohort were based on serum obtained from perchlorate workers on Tuesday morning when they had been without perchlorate exposure for > 2 days. It could be hypothesized that this lack of recent exposure had allowed recovery time that obscured some perchlorate effect. To evaluate this possibility, BMDLs for Q = 1 based on the complete data set were recalculated after replacing the workers’ Tuesday morning serum values with their Friday morning values. In both cases, the estimated effect was opposite that expected from exposure to perchlorate, and the two BMDLs were within 4% (FTI) and 50% larger (TSH) than the corresponding BMDLs based on the Tuesday morning data (Table 1). Thus, there was no indication in the data that lack of very recent exposure had allowed time for recovery.

The perchlorate doses used for the Lamm et al. study (1999) were those estimated in the original study, which used a perchlorate half-life of 8 hr compared with the 7.5 hr used for the Braverman et al. study, based on the Greer et al. study. This difference is expected to make no more than a 7% difference in the computed doses. Also, the formula used by Lamm et al. to calculate the shift doses effectively assumed an instantaneous void. More realistic assumptions would result in slightly larger estimated doses and corresponding larger BMD estimates. For example, if a 4-hr void is assumed, it is estimated that the doses would increase by approximately 10%.

Merrill et al. (2003, 2004) used data from the Greer et al. (2002) study in developing a physiologically based pharmacokinetic model for the kinetics and distribution of both iodine and perchlorate, and for the inhibition of thyroid uptake of radiolabeled iodide by perchlorate. Although the two figures are not entirely comparable, the time course of serum perchlorate in the Greer et al. study as predicted by the Merrill et al. model (Merrill et al. 2004; their Figure 6) appears to be very similar to that shown in Figure 1.

There have been three other BMD analyses for perchlorate based on human data, all of which used the Greer et al. (2002) study of perchlorate consumption by volunteers. Two of these analyses, performed by the California Environmental Protection Agency’s Office of Environmental Health Hazard Assessment (CAL/OEHHA 2004) and by the U.S. EPA (2003), modeled the ratio of the RAIU after perchlorate exposure to the baseline RAIU value, and defined the BMD as the dose corresponding to a 5% change in this ratio. The OEHHA calculated a BMDL of 0.0037 mg/kg-day, which in a 70-kg individual is equivalent to 0.26 mg/day. The U.S. EPA calculated a large number of BMDLs but emphasized some that correspond to a range of about 0.09–0.7 mg/day. These values are much smaller than the BMDLs obtained in the present analysis either from the Greer et al. study (Table 2) or from the occupational studies (Table 1). The main reason for this difference is that an RfD has traditionally been based on an adverse response, and the present analysis did not consider reduction in uptake of labeled iodine, without accompanying changes in critical thyroid parameters, to be adverse.

A third BMD analysis based on the Greer et al. study (2002) was conducted for the National Aeronautics and Space Administration by ICF Consulting (2004). BMDLs were calculated from data on FT_4_, T_4_, and T_3_. The thyroidal measurements for an individual at each of three measurement times (morning, midday, and afternoon) on day 14 minus the average of his or her measurements on two preexposure days and one postexposure day was modeled. For each of these nine cases, BMDLs were calculated corresponding to 5, 10, or 20% change in response. This resulted in 27 BMDLs that, after multiplying the average body weight in this study of 76.4 kg, range from 7 to 89 mg/day. The nine BMDLs calculated for FT_4_ correspond to a range of 8–76 mg/day, which contains the range of BMDLs for FT_4_ obtained in the present analysis both from the Greer et al. study (Table 2) and from the occupational studies (Table 1). The differences between the ICF results and those from the present analysis for FT_4_ are likely due largely to the decisions by ICF to segment the data by time of day (vs. our approach of using all the data and controlling for time of day) and to exclude the data from the uptake study. As a result, the BMDLs obtained by ICF (2004) were based on at most 22 serum values collected during exposure, whereas BMDLs for FT_4_ obtained in the present analysis (Table 2) were based on 75 such values. The ICF analysis also employed a different definition of the BMD than was used in the present analysis and employed a linear model as opposed to the nonlinear model used in the present analysis.

A more flexible dose–response model (e.g., a nonlinear model that contains a linear model as a particular case) will always result in a BMDL at least as small as that resulting from a less flexible model (e.g., a linear one). Consequently, the nonlinear model employed herein, with shape parameter K = 1, will never result in a larger BMDL than will use of a linear model (K = 1). Allowing the dose response to be highly nonlinear can also prevent the BMDL from appreciably exceeding the doses in a negative study. For example, the BMDLs calculated herein from the Greer et al. study range from 49 to 60 mg/day, whereas the largest exposure in this study was 50 mg/day. Notice also that, for the occupational studies, the values of the shape parameter, K, estimated in conjunction with the BMDL (Table 1) increase with increasing BMDL or, equivalently, as the BMDLs get closer to the highest dose in the study.

## Figures and Tables

**Table 1:** BMD estimates (mg/day) and corresponding BMDLs for perchlorate obtained from two occupational studies (Braverman et al. 2005; Lamm et al. 1999).

Thyroid indicator	*P* (0)	BMR	*Q*	BMD	BMDL	BMDL *K*^a^
All data						
FTI	0.025	0.05	0.52	67	24	1.0
	0.025	0.1	0.81	98	38	1.0
	0.025		1	118	50	1.2
TSH	0.025	0.05	0.52	INF	57	1.3
	0.025	0.1	0.81	INF	76	2.1
			1	INF	83	6.0
Eliminating influential point						
FTI	0.025	0.05	0.52	34	19	1.1
	0.025	0.1	0.81	42	27	1.5
			1	47	30	1.8
TSH	0.025	0.05	0.52	INF	32	1.9
	0.025	0.1	0.81	INF	38	4.5
			1	INF	39	6.9

Abbreviations: INF, infinite; Q, factor.

a Value of shape parameter, K, estimated in BMDL calculation.

**Table 2:** BMD estimates (mg/day) and corresponding BMDLs for perchlorate obtained from the Greer et al. (2002) study of perchlorate ingestion by volunteers.

Thyroid indicator	*P* (0)	BMR	Q	BMD	BMDL	BMDL K^a^
FT_4_	0.025	0.05	0.52	INF	49	10
	0.025	0.1	0.81	INF	52	10
			1	INF	53	10
TSH	0.025	0.05	0.52	INF	56	10
	0.025	0.1	0.81	INF	59	10
			1	INF	60	10

Q, factor.

a Value of shape parameter, K, estimated in BMDL calculation.

## References

[b1-ehp0113-001001] Braverman LE, He XM, Pino S, Cross M, Magnani B, Lamm SH (2005). The effect of perchlorate, thiocyanate, and nitrate on thyroid function in workers exposed to perchlorate long-term. J Clin Endocrinol Metab.

[b2-ehp0113-001001] Budtz-Jørgensen E, Keiding N, Grandjean P (2001). Benchmark dose calculation from epidemiologic data. Biometrics.

[b3-ehp0113-001001] CAL/OEHHA 2004. Public Health Goals for Chemicals in Drinking Water Perchlorate. Sacramento, CA:California Environmental Protection Agency, Office of Environmental Health Hazard Assessment.

[b4-ehp0113-001001] Clewell H, Lawrence G, Caine D, Crump K (2003). Determination of the occupational exposure guideline for manganese using the benchmark method. Risk Anal.

[b5-ehp0113-001001] Clewell HJ, Crump KS, Gentry PR, Shipp AM (2000). Site-specific reference dose for methylmercury for fish-eating populations. Fuel Process Technol.

[b6-ehp0113-001001] Cox DR Hinkley DV 1974. Theoretical Statistics. London:Chapman & Hall.

[b7-ehp0113-001001] Crooks J, Wayne EJ (1960). A comparison of potassium perchlorate, methylthiouracil, and carbimazole in the treatment of thyrotoxicosis. Lancet.

[b8-ehp0113-001001] Crump KS (1984). A new method for determining allowable daily intakes. Fundam Appl Toxicol.

[b9-ehp0113-001001] Crump KS (1995). Calculation of benchmark doses from continuous data. Risk Anal.

[b10-ehp0113-001001] Crump KS (2002a). Critical issues in benchmark calculations from continuous data. Crit Rev Toxicol.

[b11-ehp0113-001001] Crump KS 2002b. Benchmark analysis. In: Encyclopedia of Environmetrics (El-Shaarawi AH, Piegorsch WW, eds). Chichester, UK:John Wiley & Sons, 163–170.

[b12-ehp0113-001001] Crump KS, Van Landingham C, Shamlay C, Cox C, Davidson PW, Myers GJ (2000). Benchmark concentrations for methylmercury obtained from the Seychelles Child Development Study. Environ Health Perspect.

[b13-ehp0113-001001] Dai G, Levy O, Carrasco N (1996). Cloning and characterization of the thyroid iodide transporter. Nature.

[b14-ehp0113-001001] Gaylor DW, Slikker W (1990). Risk assessment for neurotoxic effects. Neurotoxicology.

[b15-ehp0113-001001] Godley AF, Stanbury JB (1954). Preliminary experience in the treatment of hyperthyroidism with potassium perchlorate. J Clin Endocrinol Metab.

[b16-ehp0113-001001] Greer MA, Goodman G, Pleus RC, Greer SE (2002). Health effects assessment for environmental perchlorate contamination: the dose response for inhibition of thyroidal radioiodine uptake in humans. Environ Health Perspect.

[b17-ehp0113-001001] Haddow J, Palomaki G, Allan W, Williams J, Knight G, Gagnon J (1999). Maternal thyroid deficiency during pregnancy and subsequent neuropsychological development of the child. N Engl J Med.

[b18-ehp0113-001001] ICF Consulting 2004. Recommendation for an Oral Intake Reference Dose (RfD) for Perchlorate. Prepared for National Aeronautics and Space Administration Environmental Management Division. Fairfax, VA:ICF Consulting.

[b19-ehp0113-001001] Kodell RL, Chen JJ, Gaylor DW (1995). Neurotoxicity Modeling for Risk Assessment. Regul Toxicol Pharmacol.

[b20-ehp0113-001001] Lamm SH, Braverman LE, Li FX, Richman K, Pino S, Howearth G (1999). Thyroid health status of ammonium perchlorate workers: a cross-sectional occupational health study. J Occup Environ Med.

[b21-ehp0113-001001] Lavado-Autric R, Auso E, Garcia-Velasco J, Arufe Mdel C, Escobar del Rey F, Berbel P (2003). Early maternal hypothyroxinemia alters histogenesis and cerebral cortex cytoarchitecture of the progeny. J Clin Invest.

[b22-ehp0113-001001] Merrill EA, Clewell RA, Gearhart JM, Robinson PJ, Sterner TR, Yu KO (2003). PBPK predictions of perchlorate distribution and its effect on thyroid uptake of radioiodide in the male rat. Toxicol Sci.

[b23-ehp0113-001001] Merrill EA, Clewell RA, Robinson P, Jarabek AM, Gearhart JM, Sterner TR (2004). PBPK model for radioactive iodide and perchlorate kinetics and perchlorate-induced inhibition of iodide uptake in humans. Toxicol Sci.

[b24-ehp0113-001001] NAS (National Academy of Sciences) 2000. Toxicological Effects of Mercury. Washington, DC:National Academy Press.

[b25-ehp0113-001001] NAS (National Academy of Sciences) 2005. Health Implications of Perchlorate Ingestion. Washington, DC:National Academy Press.

[b26-ehp0113-001001] Pop V, Kuijpens J, van Baar A, Verkerk G, van Son M, de Vijlder J (1999). Low maternal free thyroxine concentrations during early pregnancy are associated with impaired psychomotor development in infancy. Clin Endocrinol (Oxf).

[b27-ehp0113-001001] Shapiro SS, Wilk MB (1965). EDF statistics for goodness of fit and some comparisons. J Am Stat Assoc.

[b28-ehp0113-001001] Urbansky ET (2002). Perchlorate as an environmental contaminant. Environ Sci Pollut Res Int.

[b29-ehp0113-001001] U.S. EPA 2000a. Benchmark Dose Technical Guidance Document. (Draft) EPA/630/R-00/001. Washington, DC:Risk Assessment Forum, U.S. Environmental Protection Agency.

[b30-ehp0113-001001] U.S. EPA 2000b. Methodology for Deriving Ambient Water Quality Criteria for the Protection of Human Health. Technical Support Document Vol 1: Risk Assessment. EPA-822-B-00-005. Washington, DC:Office of Water, Office of Science and Technology, U.S. Environmental Protection Agency.

[b31-ehp0113-001001] U.S. EPA 2002. Perchlorate Environmental Contamination: Toxicological Review and Risk Characterization: NCEA-1-0503 (January 16 External Review Draft). Washington, DC:U.S. Environmental Protection Agency.

[b32-ehp0113-001001] U.S. EPA 2003. Disposition of Comments and Recommendations for Revisions to Perchlorate Environmental Contamination: Toxicological Review and Risk Characterization. External Review Draft. Washington, DC:U.S. Environmental Protection Agency.

[b33-ehp0113-001001] Venzon D, Moolgavkar S (1988). A method for computing profile-likelihood-based confidence intervals. Appl Stat.

[b34-ehp0113-001001] West R, Kodell R (1993). Statistical methods of risk assessment for continuous variables. Commun Stat Theor Meth.

[b35-ehp0113-001001] Wyngaarden JB, Wright BM, Ways P (1952). The effect of certain anions on the accumulation and retention of iodide by the thyroid gland. Endocrinology.

